# Supplementary material for: Comparative Genomic Analysis of the Marine Cyanobacterium Acaryochloris marina MBIC10699 Reveals the Impact of Phycobiliprotein Reacquisition and the Diversity of Acaryochloris Plasmids
Source: Microorganisms. 2022 Jul 7;10(7):1374. doi: 10.3390/microorganisms10071374 (PMC9324425; doi:10.3390/microorganisms10071374)
Supplement: Supplementary file 1 [file microorganisms-10-01374-s001.zip › microorganisms-1771238-supplementary.pdf]

Comparative genomic analysis of the marine cyanobacterium *Acaryochloris marina* MBIC10699 reveals the impact of phycobiliprotein reacquisition and the diversity of *Acaryochloris* plasmids.

Haruki Yamamoto[1]*, Kazuma Uesaka[2], Yuki Tsuzuki[1], Hisanori Yamakawa[1], Shigeru Itoh[3], and Yuichi Fujita[1]

Supplementaly materials

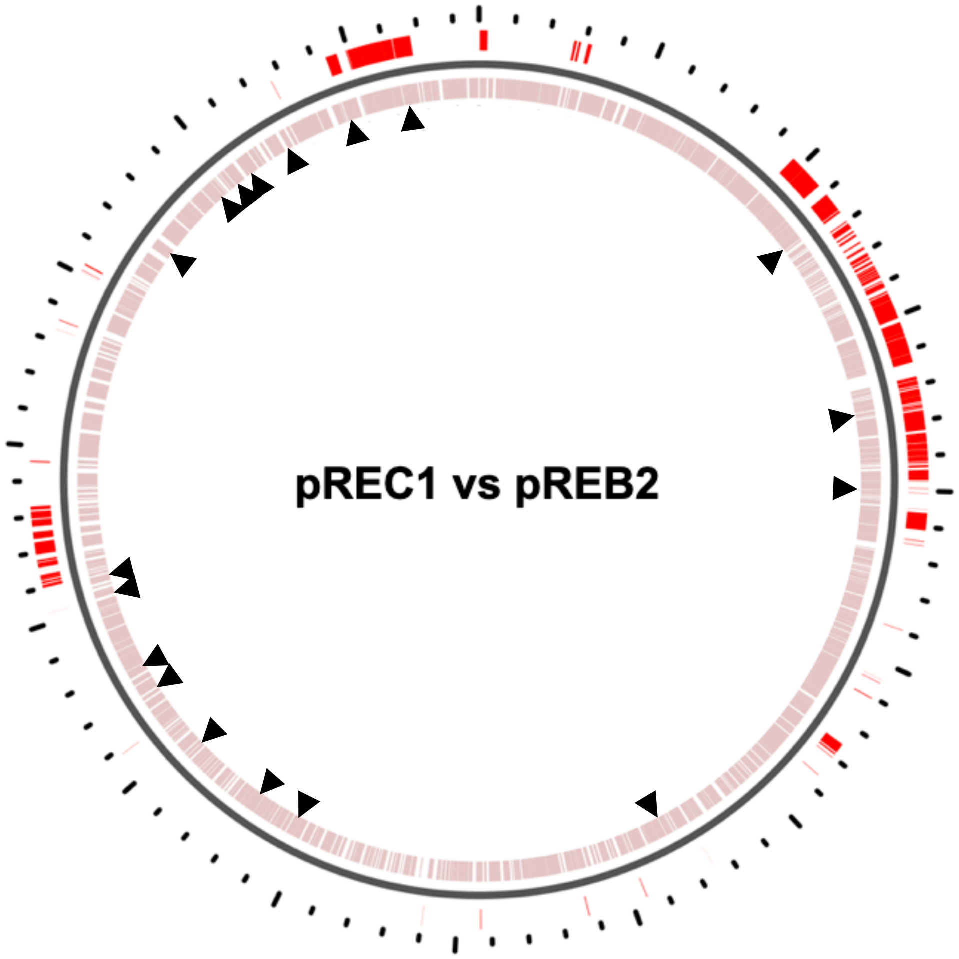

Figure S1 Loci of pREC1-specific genes in the comparison with pREC1 and pREB2.

Two plasmids with significant homology, pREC1 and pREB2, were compared and genes only present in pREC1 were mapped in red on the pREC1 sequence (light purple). Specific genes were extracted by blastn

(e-value cut off $1e^{-10}$, identity cut off 80%). Black triangles show the loci of transposase genes.

A

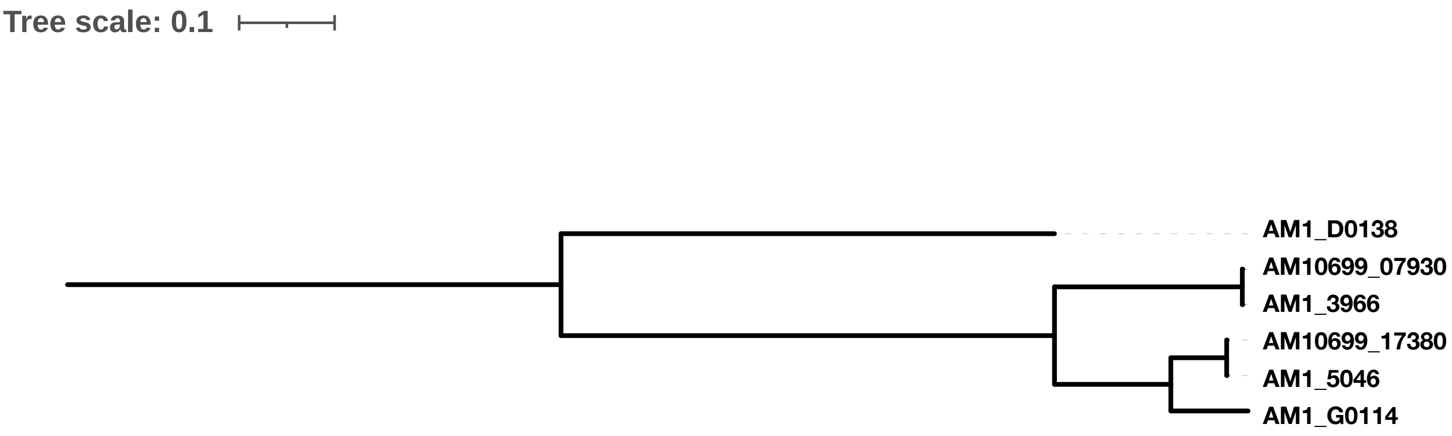

B

```
AM1_D0138         MKHFRKQLLVFGLVILTCMGMTFSFQVPVYAGEILEQPINSVQVSDPQSIP-SKESCIPI 59
AM10699_07930     MHSFVRRFGLIGLALATCLSFFG-WSQPVNALNLQS--GPTIVLADTYRNPIDEKLGTDF 57
AM1_3966          MHSFVRRFGLIGLALATCLSFFG-WSQPVNALNLQS--GPTIVLADTYRNPIDEKLGTDF 57
AM10699_17380     MRLFMRRFAVLTLVLASCLGLTG-WLQPANALDIN--------LATTYRNPVDAKLETDF 51
AM1_5046          MRLFMRRFAVLTLVLASCLGLTG-WLQPANALDIN--------LATTYRNPVDAKLETDF 51
AM1_G0114         MRLFIRRFALLTLVVASCLSLMG-WLQPVSALDIQGQSNSPGLLAAAYRNPVDAKLETDY 59
                  *: * ::: :: *.: :*:.:   :  *. * ::         ::     * . :

AM1_D0138         GEKIDLNNANALAFKDCPGYYPTLAKKIINNGPFETVDEVLNIRGLNLKQKQLLEDKLDF 119
AM10699_07930     GQKIDVNNTNVLAFMKYKGLYPTVAGKVVQNAPYSSVEDVLQIDGLTDQQKKVLQDNMGN 117
AM1_3966          GQKIDVNNTNVLAFMKYKGLYPTVAGKVVQNAPYSSVEDVLQIDGLTDQQKKVLQDNMGN 117
AM10699_17380     GQKIDLNNTNVIAFSQYKGLYPTIAGKVVQNAPYSSVEEVLNIDGLTDVQKKMLQQHLGD 111
AM1_5046          GQKIDLNNTNVIAFSQYKGLYPTIAGKVVQNAPYSSVEEVLNIDGLTDVQKKMLQQHLGD 111
AM1_G0114         GQKIDLNNTNVIAFSQYKGLYPTIAGKVVQNAPYSSVEEVLNIDGLTEPQKKLLQQHMGD 119
                  *:***:**:*.:** .  * ***:* *::*.*:.:*::**:* **.  **::*::::.

AM1_D0138         FTVSEPKTDLATRMP---P---RPMMR- 140
AM10699_07930     FTISDPDPSLVGGQDRYNPGSYKPFMK- 144
AM1_3966          FTISDPDPSLVGGQDRYNPGSYKPFMK- 144
AM10699_17380     FTITDPDPALVGGQDRYNPGAYYPVRRS 139
AM1_5046          FTITDPDPALVGGQDRYNPGAYYPVRRS 139
AM1_G0114         FTVTDPDPALVGGQDRYNTGAYYPVRRS 147
                  **:::*.  *.            *. :
```

Figure S2 Phylogenic analysis of PsbU paralogs conserved in two *Acaryochloris* strains.

A Phylogenetic tree (A) and an alignment (B) based on amino acid sequences of the two PsbUs conserved in *A. marina* MBIC10699 (AM10699_07930, 17380) and the four PsbUs conserved in *A. marina* MBIC11017 (AM1_3966, 5046, D0138, G0114). Plasmid-encoded PsbUs are AM1_D0138 (pREB4) and AM1_G0114 (pREB7).

Table S1 List of genes for the concatenated protein seaquences

A core gene search was conducted on 26 *Acaryochloris* and *Cyanothece* sp. PCC7425 and *A. thomasi* RCC1774 species, listing 897 genes that are conserved in more than 90% of their strains.

Table S2 Chromosome specific gene in *A. marina* MBIC10699

Genes encoded in the chromosome of *A. marina* MBIC10699 that are not present in the chromosome of *A. marina* MBIC11017 were extracted. Genes in red indicate specific genes that are not present in *A. marina* MBIC11017 at all, while genes in black indicate that they are present in the plasmids of *A. marina* MBIC11017.

Table S3 Chromosome specific gene in *A. marina* MBIC11017

Genes encoded in the chromosome of *A. marina* MBIC11017 that are not present in the chromosome of *A. marina* MBIC10699 were extracted. Genes in blue indicate specific genes that are not present in *A. marina* MBIC10699 at all, while genes in black indicate that they are present in the plasmids of *A. marina* MBIC10699. * indicates that the same KEGG ID was annotated in multiple genes.

Table S4 Plasmids specific gene in *A. marina* MBIC10699

Genes encoded in the plasmids of *A. marina* MBIC10699 that are not present in the plasmid of *A. marina* MBIC11017 were extracted. Genes in red indicate specific genes that are not present in *A. marina* MBIC11017 at all, while genes in black indicate that they are present in the chromosome of *A. marina* MBIC11017. * indicates that the same KEGG ID was annotated in multiple genes.

Table S5 Plasmids specific gene in *A. marina* MBIC11017

Genes encoded in the plasmids of *A. marina* MBIC11017 that are not present in the plasmids of *A*. *marina* MBIC10699 were extracted. Genes in blue indicate specific genes that are not present in *A. marina* MBIC10699 at all, while genes in black indicate that they are present in the chromosome of *A. marina* MBIC10699. * indicates that the same KEGG ID was annotated in multiple genes.
